# Assessing the influence of different alignment tools on the accuracy of a forensic epigenetic clock

**DOI:** 10.1093/bioinformatics/btag582

**Published:** 2026-08-04

**Authors:** Charlotte Sutter, Cordula Haas, Jacqueline Neubauer

**Affiliations:** Zurich Institute of Forensic Medicine, University of Zurich, Winterthurerstrasse 190, 8057 Zurich, Switzerland; Zurich Institute of Forensic Medicine, University of Zurich, Winterthurerstrasse 190, 8057 Zurich, Switzerland; Zurich Institute of Forensic Medicine, University of Zurich, Winterthurerstrasse 190, 8057 Zurich, Switzerland

## Abstract

**Motivation:**

DNA methylation (DNAm) has long been a commonly investigated biomarker in biomedical research. The current gold standard for DNAm detection is bisulfite sequencing which requires dedicated alignment tools that can handle reduced sequence complexity. One commonly used application of DNAm are epigenetic clock measurements. These clocks have been adapted by many fields for their specific needs, including forensic genetics. Here, epigenetic clocks were designed to help estimate the chronological age of a biological stain donor for investigative purposes.

**Results:**

In this study, data generated with a well-established forensic epigenetic clock is aligned with four different bisulfite-specific alignment tools: “Bwa-meth,” “Abismal,” “Bismark,” and “BS-Seeker2.” For each tool, we tested up to six different settings, altering parameters such as the maximum number of mismatches or the score function setting. The goal was to investigate whether the final predicted ages differed considerably between the tested alignment tools and settings. Quality controls such as read depth, precision, recall, F1 score, and alignment run time were also assessed. To allow other researchers to easily perform such methylation comparison analyses on their own data, a Shiny app called “MethylAge Explorer” was developed within this study. None of the tested settings for the three alignment tools “Abismal,” “Bismark,” and “BS-Seeker2” outperformed the originally used alignment tool “Bwa-meth” in terms of age prediction accuracy. However, differences in final age predictions were observed between the different alignment tools. Therefore, it is necessary to be aware of which alignment tool to use for particular epigenetic clocks.

**Availability and implementation:**

The data underlying this article and the code for the shiny app are available on GitHub (https://github.com/charlsut/methylage_explorer).

Key Messages

- Four alignment tools for bisulfite-treated DNA data were compared: “Bwa-meth,” “Abismal,” “Bismark,” and “BS-Seeker2.”
- For each alignment tool, different settings of adjustable parameters were tested.
- The accuracy of a forensic epigenetic clock was taken as the most important indication of how each alignment tool performed.
- “Bwa-meth” outperformed the other tested alignment tools in terms of age prediction accuracy.

## 1 Introduction

DNA methylation (DNAm) analysis has become one of the most widely investigated epigenetic modifications, gaining increasing importance in almost any biomedical area of research (Zhu *et al.* 2025). It is commonly associated with gene silencing as methyl groups attached to cytosines inhibit the binding of transcription factors (Zhu *et al.* 2025). Dysregulated methylation can be a cause or consequence of many influencing factors, including disease, environmental exposure, aging, and lifestyle (Horvath 2013, Kandi and Vadakedath 2015, Dhingra *et al.* 2018, Ehrlich 2019, Liu *et al.* 2023).

DNAm was recognized early on as a hallmark of aging. Changes in the methylation patterns of certain CpG sites can be used to predict a person’s biological or chronological age (Chervova *et al.* 2024). The resulting so called epigenetic clocks have been adapted for multiple purposes, from the original prediction of a person’s age to mortality prediction, prediction of disease treatment outcome, physical fitness predictions and many more (Levine *et al.* 2018, Zhu *et al.* 2019, McCrory *et al.* 2021, Sayer *et al.* 2024, Verschoor *et al.* 2024).

One area in which epigenetic clocks are being used for their own purposes is forensic genetics. The primary objective of forensic genetic analyses is to identify an individual from a biological stain, e.g. collected at a crime scene (Kayser *et al.* 2023b). The gold standard for this purpose is to generate a short tandem repeat profile and compare it with reference profiles, or with profiles in a national DNA database. However, if this comparison does not lead to a match, other tools can be used to help law enforcement to investigate the identity of an unknown stain donor (Kayser *et al.* 2023a, 2023b). These tools allow statements about the appearance of a person, including forensic age estimation. In this case, the approximate age of the stain donor is estimated at the time the stain was deposited. Forensic epigenetic clocks are designed to predict the age of a person of interest as accurately as possible in comparison to their chronological age (Kayser *et al.* 2023b).

For measuring methylation for any application, whole genome, amplicon or reduced-representation bisulfite sequencing has been the gold standard for many years (Paun *et al.* 2019, Smith *et al.* 2022). Bisulfite conversion is a chemical treatment of DNA that converts unmethylated cytosines to uracil residues, which are then converted to thymines in subsequent polymerase chain reaction (PCR) amplification steps. Methylated cytosines are protected from the treatment and remain as cytosines throughout the sequencing process (Smith *et al.* 2022). There are certain challenges associated with this conversion process. On the laboratory side of the workflow, the harsh chemical treatment often leads to DNA degradation and loss, differentiation between hydroxymethylation and methylation is not possible, and the reduced sequencing complexity can be difficult to handle for sequencing instruments (Krueger *et al.* 2012, Wreczycka *et al.* 2017, Paun *et al.* 2019). On the bioinformatics side, having a good quality alignment is of utmost importance, as it affects all downstream generated results. The primary difficulty here is the inevitable mismatch between the bisulfite converted sequences and the respective reference sequence during alignment. Not only will there be multiple mismatches, but a single site can also have different methylation states that all need to be tolerated by the alignment tool (Krueger *et al.* 2012). Another challenge in correctly aligning bisulfite-treated sequences is the ambiguous mapping of reads (Liu and Xu 2021). Because of the reduced sequence complexity, i.e. inherent in bisulfite sequencing (Kunde-Ramamoorthy *et al.* 2014), reads might map to multiple locations. As such multi-mapped reads cannot be used for any downstream analysis, it is important to have alignment tools that can minimize this multi-mapping (Liu and Xu 2021). Finally, bisulfite sequencing, especially whole genome bisulfite sequencing, requires a lot of resources such as time and computational power because of the multiple states sequencing can be in due to the conversion process (Lehle and McCarrey 2023).

Many alignment tools specifically designed for handling bisulfite-treated DNA data have been developed (Xi and Li 2009, Krueger and Andrews 2011, Guo *et al.* 2013, Li 2013, Pedersen *et al.* 2014, Chen *et al.* 2016, de Sena Brandine and Smith 2021, Farrell *et al.* 2021) and efforts have been put into comparing their performance (Gong *et al.* 2022). Since it is impossible to know the precise methylation level at any given site, there is no hard ground truth in methylation analysis. Therefore, comparisons of alignment tools usually focus on assessing parameters such as uniquely mapped reads, precision, recall, F1 score, run time, memory usage, etc.

This study takes a different approach to comparing various alignment tools by determining their performance based on the end product, in this case the accuracy of age estimates generated with a forensic epigenetic clock in comparison to the chronological age of the respective individuals. Depending on how an alignment tool calls a methylated site, slightly different methylation values (so called beta values) will be generated which in turn influence age prediction. The accuracy of age estimates is therefore the first measure by which the performance of an alignment tool will be judged. Only in a second step other measures like recall, precision, F1 score or run time will be assessed to get a better understanding of how and why the use of different alignment tools influences the final age estimates. The rationale behind this approach is that such clocks are intended to be as accurate as possible in the field of forensic genetics. Therefore, it is highly relevant if there is a difference in performance when different alignment tools are used. For this study, data generated with a published and validated forensic epigenetic clock was used (Woźniak *et al.* 2021). This tool enables the sequencing of 44 CpG sites (in eight markers), 15 of which were identified as highly age informative. The beta values of five or six of these 15 target CpG sites (depending on whether blood, buccal cells or bones are analyzed) are then used as input into the final statistical model for the respective body fluid. The recommended standard alignment tool for bisulfite converted next generation sequencing (NGS) data in this pipeline is “Bwa-meth” (Pedersen *et al.* 2014). This will be used as the “ground truth” for expected methylation values. The main aim of this study is to test other NGS-specific alignment tools using different settings for their performance: “Abismal,” which is specifically developed for faster alignment (de Sena Brandine and Smith 2021); and “Bismark” (Krueger and Andrews 2011) and “BS-Seeker2” (Guo *et al.* 2013), which are two of the current standard methylation-specific alignment tools. We compare the impact these four alignment tools have on the results of a forensic epigenetic clock. Furthermore, in order to enable end users to conveniently perform such analyses on their own data sets, we also developed a user-friendly application that performs the same analyses as described in this study.

## 2 Material and methods

### 2.1 Laboratory workflow

The laboratory workflow used for generating the data for this study was described in detail in Sutter *et al.* (2025). In brief, the study cohort consisted of venous blood samples that were collected from 102 healthy participants during blood donations at the Blood Donation Centre Zurich, Switzerland (age range [*y*] = 20.87–73.16, mean = 46.12; *n* male = 78, *n* female = 24). After DNA extraction and bisulfite conversion, sequencing was performed as published by Woźniak *et al.* (2021) on a MiSeq FGx instrument (Illumina, San Diego, USA).

This study was approved by the local Ethics Committee in Zurich, Switzerland (BASEC-No. 2023–00196) and all participants had provided a general consent at the time of blood donation to the general use of their samples for research. Only chronological age and sex were collected as meta data.

### 2.2 Summary of the general data analysis pipeline

The analysis of the raw data followed the procedure described below, independent of the alignment tool that was used. The protocol used for these experiments is an amplicon bisulfite conversion sequencing, allowing for the analysis of 44 CpG sites located in the following eight markers: *ELOVL2*, *FHL2*, *TRIM59*, *EDARADD*, *KLF14*, *MIR29B2C*, *PDE4C*, and *ASPA*.

Sequencing alignment was done on the FASTQ files with a custom Python script using one of the following four alignment tools: “Bwa-meth” (Pedersen *et al.* 2014), “Abismal” (de Sena Brandine and Smith 2021), “Bismark” (Krueger and Andrews 2011) and “BS-Seeker2” (Guo *et al.* 2013). Bam files were always created and indexed with Samtools (Li and Durbin 2009, Li *et al.* 2009). Read counts were subsequently extracted to CSV files from the bam files based on unmerged reads with the Python tool *bam-readcount* (Khanna *et al.* 2022). Beta values at the CpG sites were calculated by dividing the reads corresponding to the methylated cytosines by the sum of the methylated and unmethylated reads. Age estimates were generated with the statistical model for blood from the VISAGE software dongle, i.e. available on request from the VISAGE consortium (Kayser *et al.* 2023b).

As described in the original publication, a set of optimal markers was selected for the statistical age prediction model using stepwise linear regression with probability of F statistic, based on a statistical test of the improvement in model error (Woźniak *et al.* 2021). The effect size of the different investigated loci had been defined through a standardized regression coefficient beforehand. For the calculation of age estimates particularly from blood, six of the 15 highly age informative target sites among the total 44 CpG sites are used (Table 1, available as supplementary data at *Bioinformatics* online).

**Table 1:** Summary of tested alignment tools.^a^ e2e …end-to-end.^a^

Alignment tool	Aligner	Score function setting	No. mismatch	No. mismatch (seed)	Seed length	Sam orient.	RMSE	Abbreviation
Bwa-meth	Bwa-meth	default (Bwa-mem parameters)	–	–	–	–	3.367	Bwa_meth
Abismal	Abismal	–	−m 0.3	–	−L 250	−S	3.392	Abismal_1
Abismal	Abismal	–	−m 0.4	–	default (-L 3000)	−S	4.038	Abismal_2
Abismal	Abismal	–	−m 0.2	–	default (-L 3000)	−S	5.568	Abismal_3
Bismark	Bowtie2 (e2e)	L ,0,-1	–	−N 1	default (-L 20)	default (–directional)	4.131	Bismark_1
Bismark	Bowtie2 (e2e)	L ,0,-0.6	–	default (-N 0)	default (-L 20)	default (–directional)	4.141	Bismark_2
Bismark	Bowtie2 (local)	G,10,2	–	−N 1	default (-L 20)	default (–directional)	4.426	Bismark_3
BS-Seeker2	Bowtie2 (local)	L,0,0.1	default (-m 4)	default (-N 0)	default (-L 20)	default (–directional)	4.385	Bsseeker2_1
BS-Seeker2	Bowtie2 (local)	default (G,20,8)	−m 10	default (-N 0)	default (-L 20)	default (–directional)	4.433	Bsseeker2_2
BS-Seeker2	Bowtie2 (local)	default (G,20,8)	−m 0.2	−N 1	default (-L 20)	default (–directional)	4.795	Bsseeker2_3
Abismal	Abismal	–	−m 0.25	–	default (-L 3000)	−S	3.926	Abismal_4 (not used)
Abismal	Abismal	–	default (-m 0.1)	–	default (-L 3000)	−S	4.331	Abismal_5 (not used)
Abismal	Abismal	–	−m 0.30	–	−L 100	−S	4.760	Abismal_6 (not used)
Bismark	Bowtie2 (e2e)	L,-0.8,-0.8	–	−N 1	default (-L 20)	default (–directional)	4.132	Bismark_4 (not used)
Bismark	Bowtie2 (e2e)	L,0,-0.8	–	−N 1	−L 32	default (–directional)	4.132	Bismark_5 (not used)
Bismark	Bowtie2 (local)	default (G,20,8)	–	−N 1	default (-L 20)	default (–directional)	4.184	Bismark_6 (not used)
BS-Seeker2	Bowtie2 (local)	default (G,20,8)	−m 0.3	default (-N 0)	default (-L 20)	default (–directional)	4.393	Bsseeker2_4 (not used)
BS-Seeker2	Bowtie2 (local)	L,0.2,0.2	default (-m 4)	default (-N 0)	default (-L 20)	default (–directional)	4.397	Bsseeker2_5 (not used)
BS-Seeker2	Bowtie2 (local)	default (G,20,8)	−m 0.25	−N 1	default (-L 20)	default (–directional)	4.795	Bsseeker2_6 (not used)

a e2e... end-to-end. No. mismatch... Maximum number of allowed mismatches. No. mismatch (seed)... Maximum number of in seed alignment. Sam orient... Sam orientation reporting. RMSE... RMSE of all age predictions.

### 2.3 Alignment tools

The original publication describing this forensic epigenetic age clock used the “Bwa-meth” alignment tool. Based on a three-letter alignment strategy it wraps “Bwa-mem” (Li 2013) to map bisulfite-treated reads to a reference genome. All samples were firstly aligned with “Bwa-meth” to a custom reference sequence (available in the supplementary material of Woźniak *et al.* [2021]) using default settings (Table 1).

The second alignment tool used for this study is “Abismal” (de Sena Brandine and Smith 2021). This tool is based on a two-letter alignment approach, one for purines and one for pyrimidines. Through the reduction to two letters, it is optimized for specificity, speed and reduced memory usage for bisulfite-converted sequences. A total of six different settings were explored, modifying either the maximum number of allowed mismatches, the seed length or both (Table 1).

The third alignment tool used was “Bismark,” one of the current standard tools for whole genome bisulfite sequencing (Krueger and Andrews 2011). It is based on three-letter sequencing and uses Bowtie/Bowtie2 (Langmead *et al.* 2009, Langmead and Salzberg 2012) as the underlying aligner. For this study, six different settings of “Bismark” were used, altering the underlying aligner mode, the score function setting, the number of mismatches in the seed alignment or the seed length (Table 1).

The final alignment tool tested here was “BS-Seeker2” (Guo *et al.* 2013). It uses a three-letter alignment strategy and integrates, e.g. Bowtie2 as the underlying aligner. By using local alignment, it is designed for improved mappability in comparison to other alignment tools. Six different settings were tested on the data of this study, changing the score function settings, the maximum number of mismatches or the number of mismatches in the seed alignment (Table 1).

### 2.4 Assessing the different alignment tools

The following parameters were assessed per alignment with custom R scripts and compared between the different settings. First, base misincorporation rate was calculated by dividing the sum of adenines and guanines by the sum of nucleotides at cytosine positions. The misincorporation of adenines and guanines at such sites should be below 2%. Next, the amount of reads in all sequenced markers was assessed. In addition, overall methylation levels averaged across all 44 sequenced CpG sites were calculated. To compare the concordance of beta values across the different settings of alignment tools, mean beta value differences were calculated across all samples at each CpG site for all pairwise tool combinations.

Three accuracy parameters were assessed, namely precision, recall and F1 score. Precision in this context was defined as the proportion of detected methylated sites that were truly methylated ($\frac{\text{True}\;\text{positive}}{\text{True}\;\text{positive}+\text{False}\;\text{positive}}$), recall represented the proportion of truly methylated sites that were correctly identified as such ($\frac{\text{True}\;\text{positive}}{\text{True}\;\text{positive}+\text{False}\;\text{negative}}$) and the F1 score was the harmonic mean balancing between precision and recall, calculated as: $2\times \frac{\text{precision}\times \text{recall}}{\text{precision}+\text{recall}}$. As all three parameters require a “true value” against which to compare the generated beta values for each alignment setting with, the original “Bwa-meth” alignment was defined as the ground truth.

Finally, the run time required to complete the alignment for each setting was summarized and compared.

Statistical comparisons and graphical representations were performed with the R packages stats, ggplot2, and tidyverse (Wickham 2016, Wickham *et al.* 2019, R Core Team 2023) using the functions *kruskal.test* and *pairwise.wilcox.test* with Benjamini–Hochberg correction.

As the focus of this study was the effect of different alignments on the final outcome of the analysis pipeline, namely the estimated chronological age, the errors in the estimated ages compared to the actual chronological ages were used to measure how well each alignment tool performed with the different settings. For this purpose, the route mean squared error (RMSE) was calculated across the entire sample cohort for each alignment setting using the following formula: $\sqrt{\mathrm{mean}\left({\left(\text{actual}-\text{predicted}\right)}^{2}\right)}$.

### 2.5 MethylAge Explorer: a new shiny app for comparing methylation data

A new shiny app in R called “MethylAge Explorer” was developed to enable users to perform comparisons as the ones described above on their own data. Similar to the results presented in this study, this shiny app requires data from any kind of bisulfite sequencing, where each sample has at least two different sets of methylation beta values. For example, using the data presented in this study, each tested alignment generates its own set of beta values, resulting in up to 19 different groups that can be compared (Table 1). However, the shiny app is not limited to testing differences resulting from different bioinformatic pipelines, it also allows for the comparison of biologically distinct methylation values. Specifically, if for instance methylation data was generated from two different body fluids from the same individual, the results of these two measurements can also be compared with this shiny app.

In short, this shiny app allows users to compare age predictions, read depth, base misincorporation, concordance, mean methylation, accuracy parameters (precision, recall and F1 score) as well as run time. A more detailed documentation on the required input files and how to operate this application can be found on the front page of the app’s interface.

## 3 Results

From the six settings tested for each alignment tool, three were selected: the setting yielding the lowest RMSE for each alignment tool, the setting with the highest RMSE and one setting in between. These nine settings, in addition to the originally used “Bwa-meth,” were analyzed and compared between one another, except for the age prediction results, for which all 19 alignment settings will be reported. All settings were given an abbreviation i.e. also used in the following analyses (Table 1).

### 3.1 Age prediction

**Figure 1:**
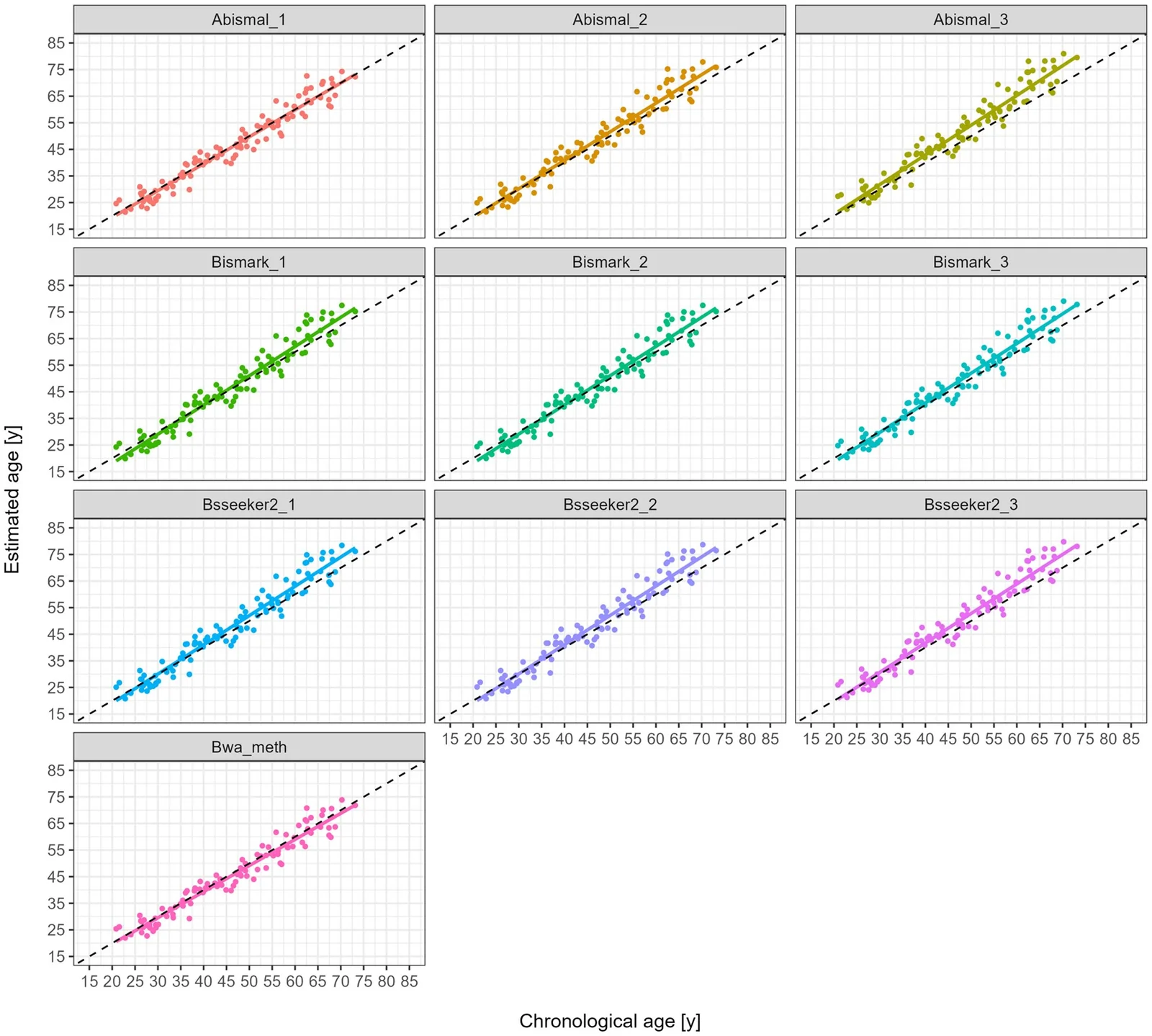
Age prediction results for the 10 alignment settings. Details about the different settings can be found in Table 1. The black dotted line represents the line of identity and the colored lines represent the trend lines. The estimated and the chronological ages are depicted in years [y].

Age prediction was assessed with the RMSE value that ranged from 3.393 to 5.568 years across the nine different new alignment settings (Table 1 and Fig. 1). The reference RMSE value for the “Bwa-meth” alignment tool was 3.367 years. A Kruskal–Wallis test comparing the age prediction errors revealed no statistically significant results (*P*-value = .10). The age prediction results for all 19 tested alignment settings are depicted in Fig. 1, available as supplementary data at *Bioinformatics* online.

### 3.2 Read counts, base misincorporation and mean methylation

**Figure 2:**
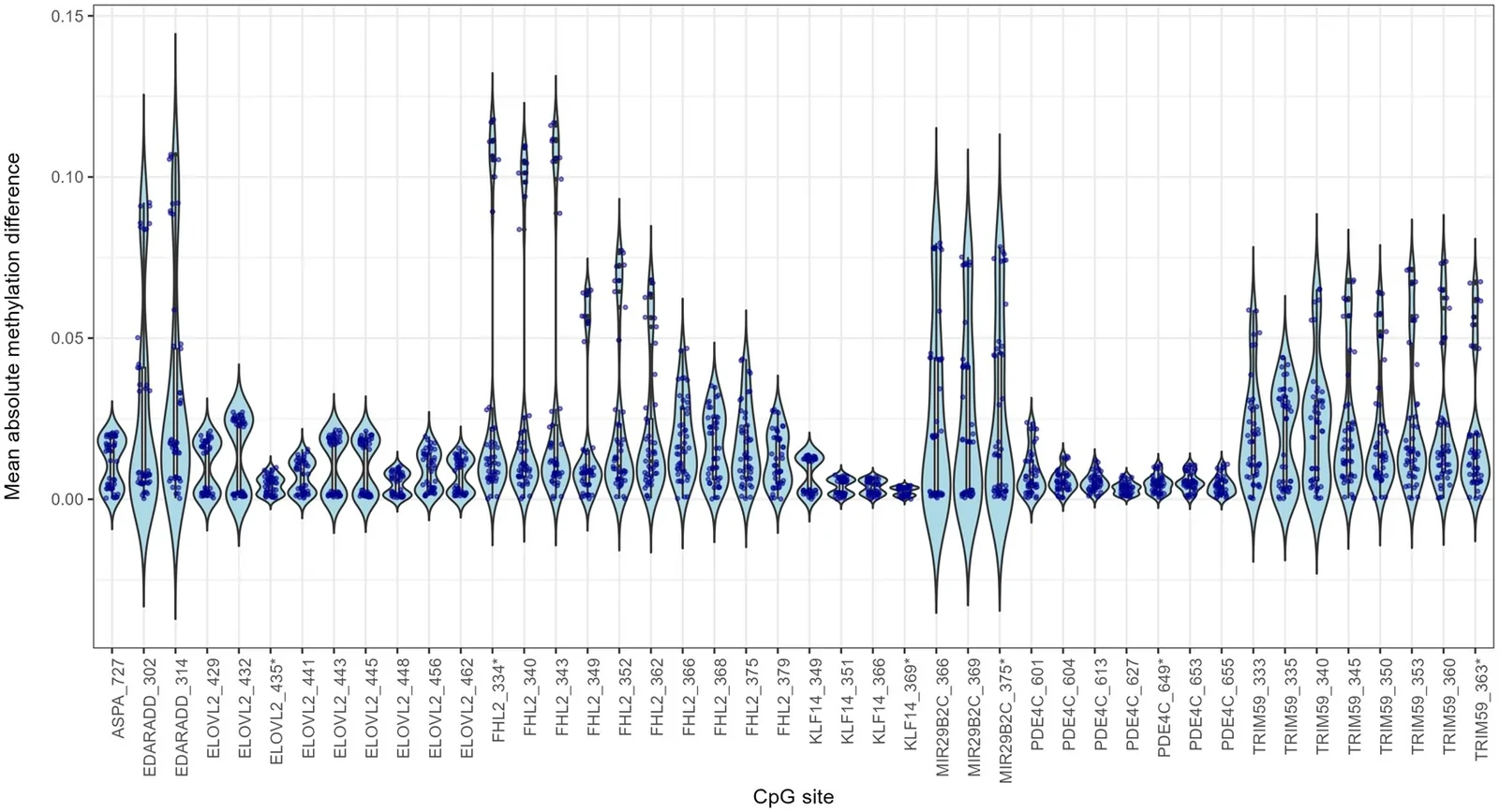
Concordance of beta values between tested alignment settings presented in a violin plot. Pairwise absolute differences between alignment settings were calculated, averaging across all samples and displayed for each sequenced CpG site. The six target CpG sites used for age prediction from blood are marked with (*).

The total number of reads for the 15 highly age informative target CpG sites was visualized for all 10 alignment settings, while base misincorporation rate was only visualized for the six CpG sites used in the age prediction statistical model of the here used forensic epigenetic blood clock (Woźniak *et al.* 2021; (Figs. 2 and 3, available as supplementary data at *Bioinformatics* online). Interestingly, the read count analysis showed that especially the settings Abismal_1 and Abismal_2 often generated higher read counts than the other alignment settings. For the base misincorporation, especially the target site in *KLF14* showed varying misincorporation rates of up to 2%. For a statistical comparison, the sum of all reads per sample and alignment setting was calculated and compared with pairwise Wilcoxon tests (Table 2, available as supplementary data at *Bioinformatics* online). Base misincorporation rates were averaged across all six CpG sites per sample and alignment tool and statistically compared as well (Table 3, available as supplementary data at *Bioinformatics* online). Interestingly, almost all comparisons were statistically significant.

Mean beta values across all 44 sequenced CpG sites were calculated per sample and alignment setting. The original “Bwa-meth” alignment setting yielded 0.371 as mean beta value, while for the other alignment settings, mean overall beta values ranged from 0.343 to 0.370 (Table 4 and Fig. 4, available as supplementary data at *Bioinformatics* online). Interestingly, the mean overall beta values were highest for the “Bwa-meth” alignment tool. Statistical comparisons were again performed with pairwise Wilcoxon tests, using the mean beta values per sample as input (Table 5, available as supplementary data at *Bioinformatics* online). Only 19 out of 45 pairwise comparisons yielded significantly different mean beta values.

### 3.3 Concordance

Concordance between the beta values of the different alignment settings was assessed by calculating the mean absolute difference between each pair of alignment settings across all samples for each of the 44 sequenced CpG sites (Table 6, available as supplementary data at *Bioinformatics* online). The CpG sites in *MIR29B2C*, *FHL2*, *EDARADD*, and *TRIM59* showed overall higher mean beta value differences in pairwise comparisons of alignment settings than the CpG sites in *ASPA*, *ELOVL2*, *KLF14*, and *PDE4C* (Fig. 2 and Table 7, available as supplementary data at *Bioinformatics* online).

### 3.4 Accuracy parameters

Commonly used accuracy parameters to assess the performance of an alignment tool were used for this study as well. Due to the lack of a true ground truth, the “Bwa-meth” results were used as reference against which to calculate precision, recall and F1 score. Precision was particularly high for the “Bismark” alignment settings while recall was also high for the “Abismal” settings (Fig. 5, available as supplementary data at *Bioinformatics* online). Summaries of all parameter values and statistical comparisons are summarized in Tables 8 and 9, available as supplementary data at *Bioinformatics* online. Interestingly, recall was not statistically different between any alignment setting comparison. In addition, almost no comparisons of F1 scores showed significant differences between alignment settings. Contrarily, precision was significantly different for about two-thirds of the comparisons.

### 3.5 Run time

Run time was recorded using Python’s *time.time()* command before and after the alignment run command, printing wall-clock elapsed time in seconds to log files. While the “Bwa-meth” alignment only took an average of 15.96 s per sample for aligning (median = 15.45 s), the “Abismal” settings took on average between 17.26 and 19.13 s (median between 18.39 and 20.48 s), the “Bismark” settings took between 37.27 and 98.01 s (median between 31.00 and 108.00 s) and the “BS-Seeker2” settings took between 75.28 and 94.26 s (median between 83.46 and 103.39 s; Table 10 and Fig. 6, available as supplementary data at *Bioinformatics* online). Statistical comparisons revealed that most pairwise comparison were significant. They are summarized in Table 11, available as supplementary data at *Bioinformatics* online.

### 3.6 Shiny app

A shiny app called “MethylAge Explorer” was developed in R and its code is available on GitHub (https://github.com/charlsut/methylage_explorer). It includes a front page with instructions for users on which files to upload. There is the possibility to upload a methylation data CSV file containing read counts, sample IDs and which sample belongs to which group or set of methylation value, respectively. A separate file containing run time values if comparing alignment tool performances can be uploaded as well, but is optional. Generated plots and tables can be downloaded from the shiny app. Plot dimensions can be set manually according to the users’ needs and tables will be downloaded as CSV files. Warning messages are incorporated in case data is missing or the formatting of the input file is not set according to the instructions. A mock data set input file and the file for the data presented in this study is provided on the GitHub page for reference as well.

## 4 Discussion

This study tested whether different alignment tools for methylation calling affect how accurately a forensic epigenetic clock predicts the chronological age of the respective individuals. For this purpose, data was used that had been generated with a published forensic epigenetic age clock (Woźniak *et al.* 2021). In this workflow, “Bwa-meth” (Pedersen *et al.* 2014) is the standard alignment tool and results obtained through alignment with this tool served as reference in this study. Three methylation specific alignment tools were newly tested, namely “Abismal” (de Sena Brandine and Smith 2021), “Bismark” (Krueger and Andrews 2011), and “BS-Seeker2” (Guo *et al.* 2013) tweaking parameters such as the maximum allowed mismatch rate, the score function settings, or the seed length. This strategy should reveal whether and how adjusting alignment tools and settings influences the final age prediction results of a given study cohort. For a forensic epigenetic clock, this could be highly relevant because the objective of such a clock is to provide the most accurate estimate of a person’s chronological age. If these results are affected by choosing a different alignment tool or settings, it is important to ensure that no alignment tools/settings are used that increase the age prediction errors. However, if it is potentially possible to reduce the prediction errors, this would also be relevant information for the forensic genetics community.

The first striking observation of this study was that the different new alignment tools produced RMSE values close to those of the original “Bwa-meth” alignment, but none of them outperformed it in terms of age prediction accuracy. The “Bwa-meth” alignment achieved an RMSE of 3.367 years in this cohort, and the closest result to this was achieved with the “Abismal” alignment tool allowing 30% mismatches and with a seed length of 250 (RMSE = 3.392 years). Conversely, the “Abismal” tool showed the highest RMSE value (5.568 years) of all tested alignment tools as well, this time when allowing for only 20% mismatches and with a default seed length of 3000. Possibly the difference in seed length between Abismal_1 and Abismal_3 (250 versus 3000) was one of the reasons why in those two settings the lowest and highest RMSE value of all new alignment settings was found. For clinically relevant epigenetic clocks, an RMSE difference of approximately 2 years between the alignment setting with the highest and the lowest RMSE might not seem big enough to report. However, for forensic epigenetic clocks, it is indeed noteworthy whether the RMSE value is five or only 3 years. Here, an epigenetic clock that produces an RMSE of 5.568 in blood might nowadays be considered inferior to other forensic age clocks. In conclusion, alignment settings such as “Abismal_3” should not be used in combination with this epigenetic clock workflow for forensic applications.

As none of the here tested alignment settings surpassed the original “Bwa-meth” alignment tool in terms of age prediction accuracy, it was investigated whether technical parameters such as read counts, precision, recall, concordance, run time, etc. differed between the tested alignment settings. The rationale was to identify whether any of the tested alignment settings had technical advantages over the original “Bwa-meth” that would make them preferable to the original alignment tool although age prediction was not more accurate. Of the 18 tested new alignment settings (six per alignment tool) only nine were thoroughly analyzed for differences in technical parameters. This reduction was based on the RMSE values of each setting, taking the best, the worst and an in-between performing setting per alignment tool. The rationale behind this selection was to demonstrate whether settings that produced low RMSE values compared to those producing comparably higher RMSE values differed in technical measures as described below.

Cumulative read counts at the sequenced eight markers were predominantly comparable. Only two of the three “Abismal” settings stood out as they showed higher read counts for most markers (Fig. 2, available as supplementary data at *Bioinformatics* online). This was possibly a result of allowing 30% and 40% mismatches compared to 4%–20% mismatches with the other tools/settings, which caused more reads to pass. When investigating the base misincorporation rate at the six target CpG sites of the blood age prediction model used in this study, it was particularly noteworthy that there was a higher variation in the *KLF14* target site. Interestingly, the highest misincorporation rate at this site of more than 2% was observed in the “Abismal_3” setting, notably the tested alignment with the highest RMSE value. The second highest overall base misincorporation rate was found in “BS-Seeker2_3”, the setting with the highest RMSE value within the “BS-Seeker2” settings. Possibly this could be a hint that alignment settings with higher RMSE values are correlated with higher base misincorporation rates. Another investigated parameter was the overall mean beta value per alignment setting. Interestingly, within each of the newly tested alignment tools, the setting with the highest RMSE value always had the highest overall mean beta value. However, due to the limited numbers of CpG sites investigated here it is difficult to say whether this is a purely coincidental finding or whether it has some actual meaning.

As it was difficult to draw meaningful conclusions from the overall beta value analysis, a more in depth analysis was performed by looking at the concordance between mean beta values across all samples of all possible pairs of alignment settings at each sequenced CpG site. In four of the eight markers (*ASPA*, *ELOVL2*, *KLF14*, and *PDE4C*), all CpG sites showed very little absolute beta value differences between any of the pairwise comparisons. In the remaining four markers (*EDARADD*, *FHL2*, *MIR29B2C*, and *TRIM59*), mean beta value differences were partially as high as 0.125. Considering that the strongest age predictive marker, *ELOVL2*, was among the markers with very high concordance in any pairwise comparison of alignment tools, this might explain why there were no significant differences in age prediction errors between the tested alignment settings. Markers such as *FHL2* and *MIR29B2C* contribute less to the final age prediction result and their lower concordance between alignment settings will therefore not affect the final result considerably.

In a next step, accuracy parameters commonly used for assessing the performance of alignment tools (Gong *et al.* 2022) were investigated. These included precision, recall and F1 score. Methylation analysis is more complex than genetic analysis as there is no clearly defined reference base at each genomic position. For this study, the beta values generated with the standard “Bwa-meth” alignment were therefore defined as ground truth against which all parameters were compared. Particularly high precision was achieved with the “Bismark” alignment tool, which can be seen from the fact that most of the reads detected as methylated by “Bismark” were also methylated in “Bwa-meth.” Recall was consistent across all tested alignment settings, meaning that most reads detected as methylated by “Bwa-meth” were also identified as such by the other settings. The F1 score, a balanced parameter between precision and recall was also very comparable across all new alignment settings.

The final parameter investigated was the run time required for running the alignment. Interestingly, “Bwa-meth” had the smallest mean run time with 15.86 s, closely followed by the “Abismal” settings with approximately 17 and 19 s. The “Bismark” settings with the lowest and an in-between RMSE value had mean run times of approximately 30 and 37 s, while the setting with the worst RMSE had a three-times increased run time of approximately 98 s per sample. All “BS-Seeker2” settings also performed badly in terms of run time with 94.26, 75.28, and 90.74 s, respectively. Although these differences in run time are not crucial for a study cohort consisting of only 102 samples, it might be a consideration for larger cohorts not to use the “BS-Seeker2” settings and the “Bismark_3” setting.

Taken all findings together, “Bwa-meth” is still the best alignment tool to use for this forensic epigenetic age clock. None of the tested new alignment tools outperform “Bwa-meth” with regards to RMSE values. Additionally, none of the tested technical parameters offer a reason to choose a different alignment tool. Read counts, base misincorporation, mean methylation, and concordance are comparable across all alignment settings. These are not parameters on which a decision for or against an alignment tool is based. Such parameters would rather be recall, precision, F1 score, and run time. However, the lack of an actual ground truth for precision, recall, and F1 score are an important limitation for the usefulness of these parameters in this study. If we were to consider only these parameters in a decision-making process, it might be possible to say that the “Bismark” settings are preferable because of their high precision. However, as the original “Bwa-meth” alignment is defined as ground truth, a comparison between the new alignment settings and “Bwa-meth” is not possible, which diminishes the usefulness of these parameters for assessing whether a new alignment tool could outperform the original alignment tool. Finally, the mean run time is of particular importance when handling large data sets with far more than 100 samples as in this study. However, even “Abismal,” a tool that was especially designed for fast alignment by using a two-letter alignment approach (de Sena Brandine and Smith 2021), was not faster than “Bwa-meth” in our cohort.

Although the comparison of different alignment tools and different settings in this study only revealed that the original alignment tool is still the best for this particular forensic epigenetic clock for the use in a forensic context, other methylation-based applications in different fields might benefit from a comparison of alignment tools. To facilitate such comparisons for other users, we designed the R shiny app “MethylAge Explorer.” This app allows the user to perform all analyses in the same way as described in this article. However, it is not restricted to test different alignment tools on the same samples, but this app can be useful for any application as long as the same sample has at least two different methylation profiles. When comparing alignment tools, this would mean uploading CSV files with beta values for the different alignment settings for all samples. If, e.g. methylation data has been collected from blood and saliva, this app is also able to analyze differences between blood and saliva methylation profiles as long as they are from the same person. This app was specifically designed for anyone without the resources or the time to perform extensive analyses such as professionals working in forensic casework or clinical diagnostic laboratories.

## 5 Conclusion

This study revealed that none of the settings of the here tested alignment tools “Abismal,” “Bismark,” or “BS-Seeker2” increased the age prediction accuracy of a forensic epigenetic clock (Woźniak *et al.* 2021). As technical parameters did not provide a convincing reason why any of the newly tested alignment tools would be preferable for the use with this forensic epigenetic clock, we concluded that “Bwa-meth” still is the best alignment tool for this epigenetic clock. There are other methylation specific alignment tools that could have been tested in this study. However, given our current findings, it seems unlikely that any other alignment tool could outperform “Bwa-meth” in terms of the most important parameter for a forensic epigenetic clock: age prediction accuracy. The newly created shiny app “MethylAge Explorer” shall facilitate and accelerate future studies for similar investigations of methylation data.

## Supplementary Material

btag582_Supplementary_Data

## Data Availability

The data underlying this article and the code for the shiny app are available on GitHub (https://github.com/charlsut/methylage_explorer).
