# Supplementary material for: Assessing the influence of different alignment tools on the accuracy of a forensic epigenetic clock
Source: Bioinformatics. 2026 Aug 4;42(8):btag582. doi: 10.1093/bioinformatics/btag582 (PMC13471995; doi:10.1093/bioinformatics/btag582)
Supplement: Supplementary file 1 [file btag582_supplementary_data.zip › Alignment_paper_Supplements.pdf]

Supplementary material: Assessing the influence of different alignment tools on the accuracy of an epigenetic clock

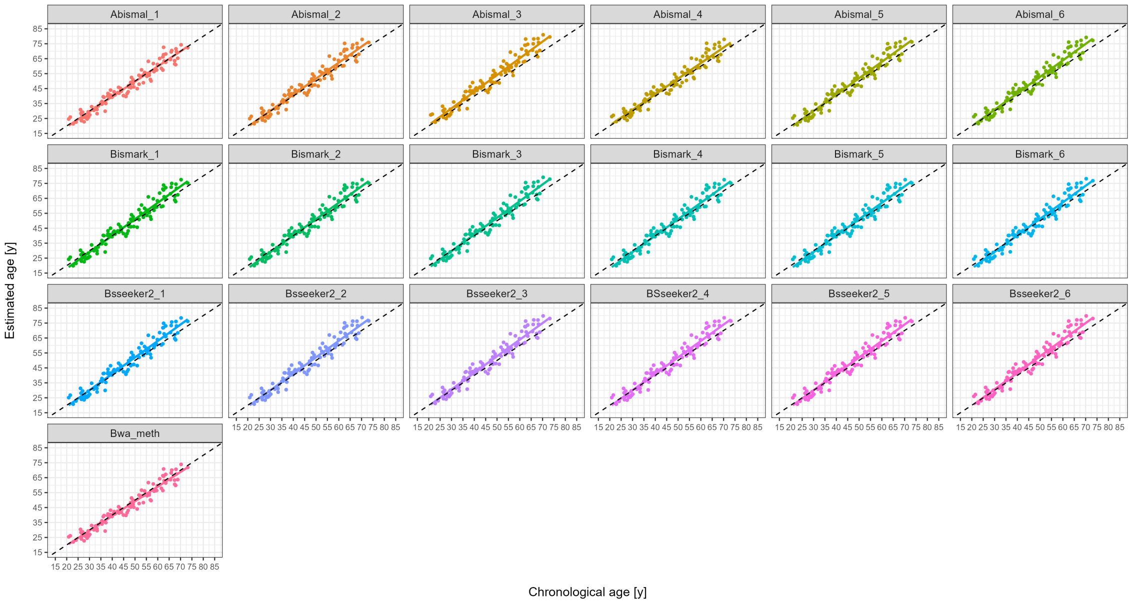

Supplementary figure 1: Age prediction results for all 18 tested alignment settings (and the original ‘Bwa-meth‘). Explanations of the abbreviations can be found in Tabel 1 in the main article. The black dotted line represents the line of identity and the colored lines represent the trend lines. Estimated and chronological ages in years.

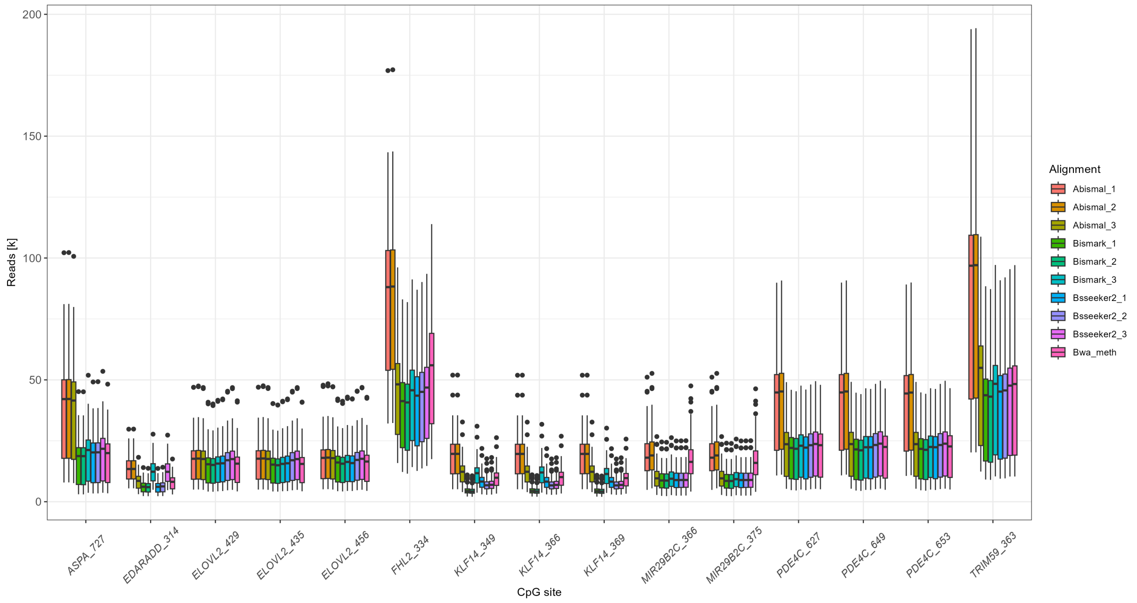

Supplementary figure 2: Read counts summarized for the 15 highly age informative CpG sites for all alignment settings.

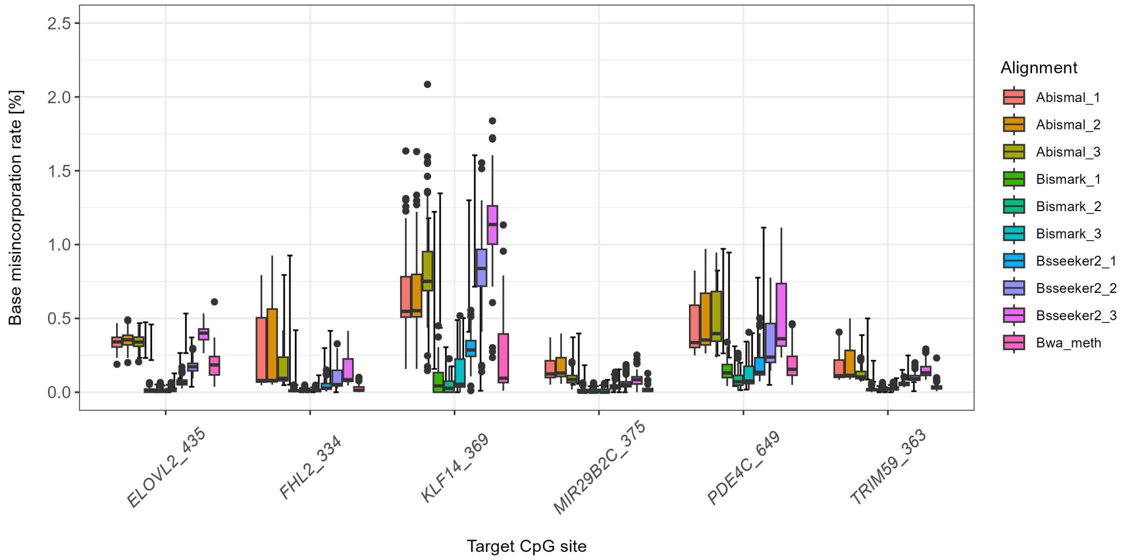

Supplementary figure 3: Base misincorporation [%] per blood target CpG site for all alignment settings.

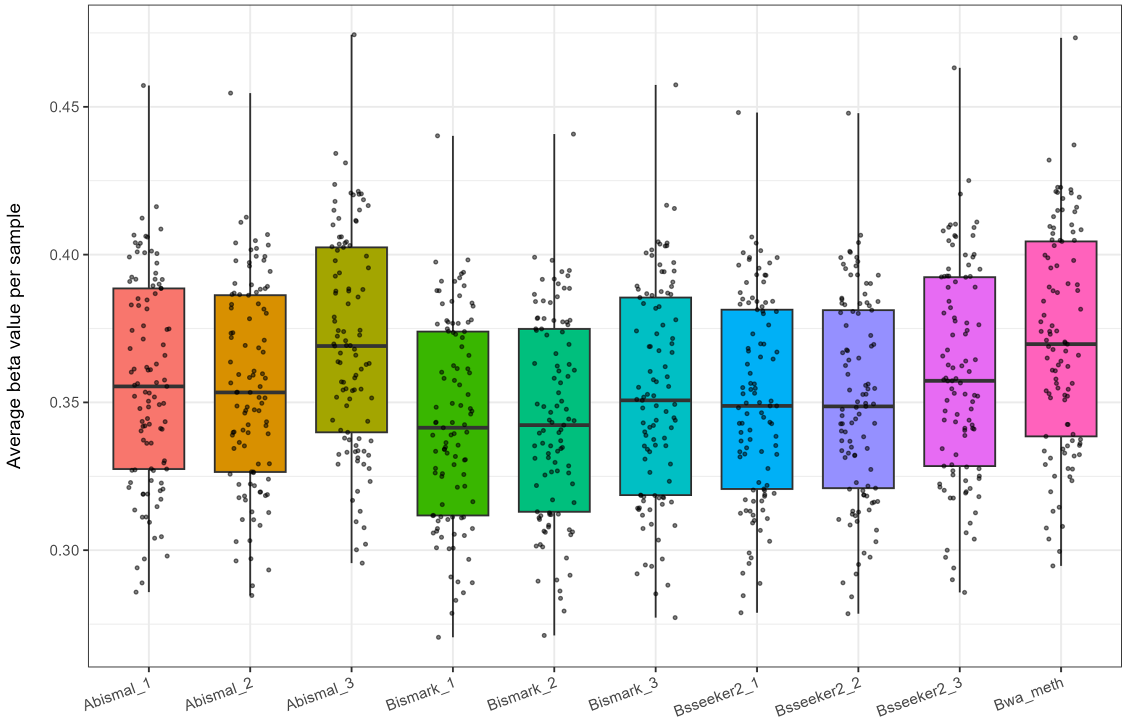

Supplementary figure 4: Mean methylation beta values per alignment setting, calculated as mean beta value across all 44 CpG sites.

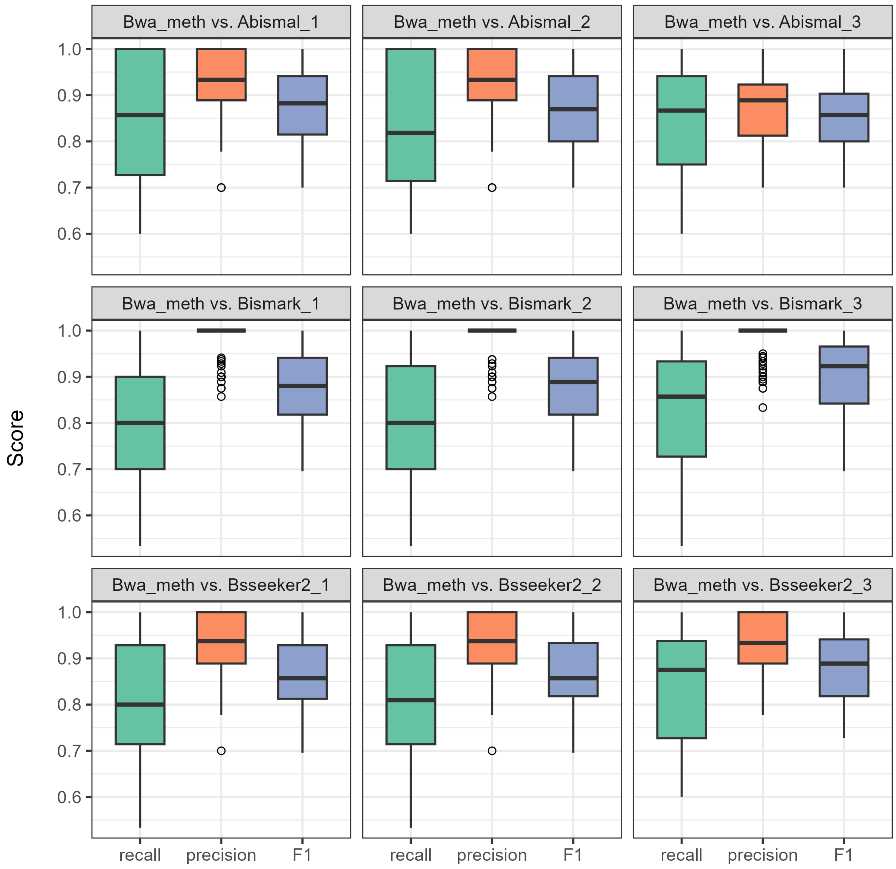

Supplementary figure 5: Precision, recall and F1 score taking Bwa-meth as ground truth per comparison.

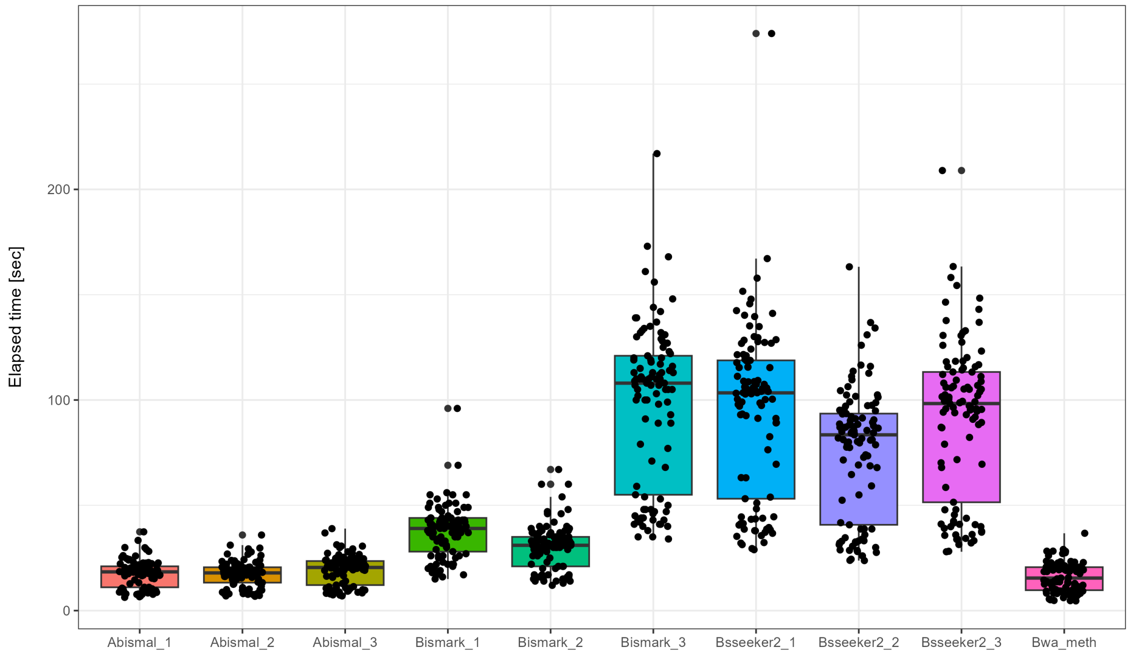

Supplementary figure 6: Elapsed run times of alignment commands in sec per alignment setting.
